# Challenges to increase the AI and ET markets in Brazil

**DOI:** 10.21451/1984-3143-AR2019-0050

**Published:** 2019-10-22

**Authors:** Pietro Sampaio Baruselli, Bruna Lima Chechin Catussi, Laís Ângelo de Abreu, Flavia Morag Elliff, Laísa Garcia da Silva, Emiliana de Oliveira Santana Batista

**Affiliations:** Departamento de Reprodução Animal, Faculdade de Medicina Veterinária e Zootecnia, Universidade de São Paulo, São Paulo, SP, Brasil.

**Keywords:** Artificial insemination, embryo transfer, synchronization, reproductive efficiency, economic return

## Abstract

Artificial insemination (AI) and embryo transfer (ET) are the most widely used biotechnologies in the world with the goal of increasing genetic gain and improving reproductive efficiency of beef and dairy herds. The protocols for ovulation synchronization for timed AI (TAI) or ET (TET) are tools that allow artificial insemination or transfer of a high number of embryos in a pre-established moment and without the necessity of estrous detection. Currently, 86% of inseminations in Brazil are performed using TAI (13.6 million TAI out of a total of 15.4 million doses of semen marketed in 2018). With the use of TAI, it was possible to verify that the percentage of artificially inseminated females in Brazil went from 5.8% in 2002 to 13.1% in 2018. The ET market also presented considerable growth in the last 20 years. There was an increase of approximately 8 fold in the number of produced embryos, escalating from 50,000 in 1999 to 375,000 in 2017. In this period, there was a significant increase on the *in vitro* embryo production, which represented 92.1% of embryos produced in Brazil in 2017. Also, in this period, there was an increase on the embryo production of dairy breeds and reduction on the embryo production of zebu breeds in comparison to data from the early 2000’s. TET increases significantly the number of recipients suitable to receive an embryo. After synchronization, 75 to 85% of recipients present a suitable CL for ET without estrous detection. Currently, many synchronization and resynchronization protocols for TAI/TET have been studied to attend different managements, breeds and animal categories, with predictable and satisfactory results. With the intensification of the use of these biotechnologies, it is possible to obtain elevated reproductive efficiency with increase on the genetic gain, which determines greater productivity and economic return for dairy and beef farms. However, the challenge to keep the market growing in the next decade could depend on some factors, such as: increase of the extension services for producers and of the extension training for specialists, improvement of the technological advances to develop more efficient and cost-effective products and practical protocols, increase the integration between universities, research institutes, veterinarians and industries and also, asses market demand for production of animal protein with higher quality, efficiency and environmental and economic sustainability.

## Introduction

The accelerated growth of the world population is generating a significant increase in the demand for food, causing concern for the production of animal proteins to meet the growing number of people on the planet (FAO, 2017). In this context, Brazil is relevant because it is the fifth largest country in territorial extension and has the largest commercial cattle herd in the world (221.81 million heads, IBGE, 2018).

In 2017, the number of bovine slaughters in Brazil reached 39.2 million, with an estimated production of 9.71 million tons of carcass equivalent, representing 14.4% of world meat production (ABIEC, 2018). Despite this positive scenario, Brazilian beef cattle production still has low production efficiency, and ranks second in the world classification of meat production, led by the United States, which produce 17.9% of the world meat production (ABIEC, 2018).

The national production of fluid milk was 33.5 billion liters, out of a total of 17 million milked cows, corresponding to the yield of 1,943 liters of milk per cow per year (IBGE, 2017). In this context, each animal contributed with only 5.4 liters of milk produced per day, evidencing the low efficiency of this activity in Brazil (IBGE, 2017). These numbers rank Brazil as the fourth largest milk producer (falling behind the United States, India and China), despite having the world's largest cattle herd. Also, Brazil is not self-sufficient in the production of cattle milk, and in this scenario, importation is necessary to supply the domestic market (IBGE, 2017).

Thus, it is essential to develop and improve technologies that collaborate with increasing productivity on farm, optimizing the breeding systems and the profitability of the herds. Among the developed technologies, reproduction biotechnologies are noticeable.

Artificial insemination (AI) is the most widely used reproductive biotechnology in the world and its application brings great benefits to the herds when compared to the use of natural service (Lima *et al*., 2010; Lamb and Mercadante, 2016; Baruselli *et al*. al., 2018a). The technique allows the use of the semen of genetically superior bulls, accelerating the genetic gain and resulting in more productive calves, which generate greater economic return to the meat and milk producer (Baruselli et al., 2017b). In addition, AI prevents the transmission of venereal diseases (Vishwanath, 2003) and allows better control of the herd, increasing the uniformity of calves when compared to natural service (Rodgers *et al*., 2015; Baruselli *et al*., 2017a).

In order to facilitate the use of AI in rural properties, timed artificial insemination (TAI) was developed (Pursley *et al*., 1995). This reproductive biotechnology eliminates the necessity of estrous detection and allows anestrous cows to be inseminated, increasing the reproductive efficiency of cows and heifers (Rhodes *et al*., 2003). Furthermore, the use of TAI anticipates and concentrates conception at the beginning of the breeding season, increasing the reproductive and productive efficiency of farms (Baruselli *et al*., 2002; Baruselli et al., 2004; Bo et al., 2007; Sá Filho *et al*., 2013; Sartori *et al*., 2016).

Embryo transfer (ET) allows the dissemination of high value genetic material from both males and females, increasing the genetic gain of animal breeding programs when compared to AI. Furthermore, ET associated with the advent of genomic technology and endocrine markers (AMH) allows the use calves as oocyte donors to produce embryos from young cattle, which is an important strategy to accelerate genetic gain by decreasing generation intervals (Batista et al., 2016). The ET has presented considerable growth in the last decades, mainly due to the scientific and technological development of innovative processes of embryo production. Currently, *in vitro* embryo production (IVEP) represents 92.1% of the embryos produced in Brazil, and 66% of the embryos produced in the world (Viana *et al*., 2018; IETS, 2018). In addition to the increase on the IVEP, there was the development of synchronization techniques for fixed-time embryo transfer (TET), which increases the number of recipients suitable for receiving an embryo and eliminates the necessity for estrous detection, allowing the establishment of this biotechnology in beef and dairy farms (Baruselli *et al*., 2000; Bo *et al*., 2002; Baruselli *et al*., 2010).

## The evolution of artificial insemination

The Brazilian market for artificial insemination traded approximately 7.0 million doses of semen in 2002. In 2018, this market reached 15.4 million semen doses marketed (ASBIA INDEX, 2019), with a 220% growth in that period. Still, compared to the previous year (2017 to 2018), the semen market grew 13.7%. For the calculation of the number of semen doses marketed in Brazil, data from the ASBIA INDEX were considered (representing 90% of the Brazilian semen market), with adjustments to 100% of the market (Baruselli *et al*., 2019a). These data clearly demonstrate that artificial insemination has gained ground in Brazil over the years.

**Figure 1:**
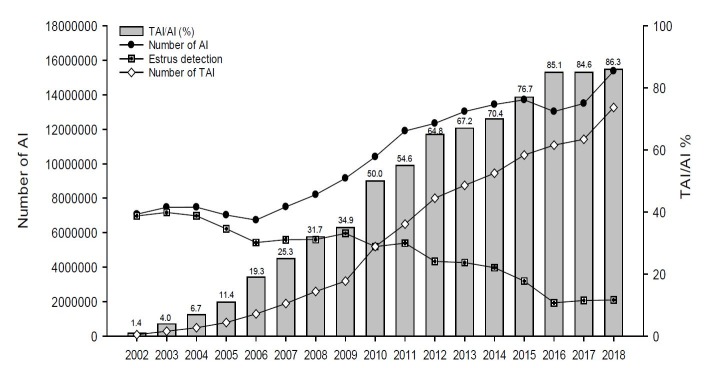
Evolution of timed artificial insemination (TAI) or artificial insemination with estrous detection in cattle in Brazil (adapted from Baruselli, 2019a).

The increase in the AI market in Brazil occurred simultaneously with the introduction of TAI technology. In 2002, according to data obtained by the Animal Reproduction Department of FMVZ/USP (Baruselli *et al*., 2012), the number of protocols marketed was 100,000, which shows that only 1% of the inseminations in Brazil were performed by TAI that year. In 2018, the number of TAI reached 13.3 million procedures, indicating that 86% of the inseminations were performed using TAI in Brazil (Baruselli 2019a; Fig. 1). The TAI market growth in 2018 also showed a significant increase of 16.1% when compared to 2017 (11.4 million TAI). It is possible to verify that the TAI has grown 130 fold in the last 16 years, bringing great advances and benefits to the entire meat and milk production chain.

**Figure 2:**
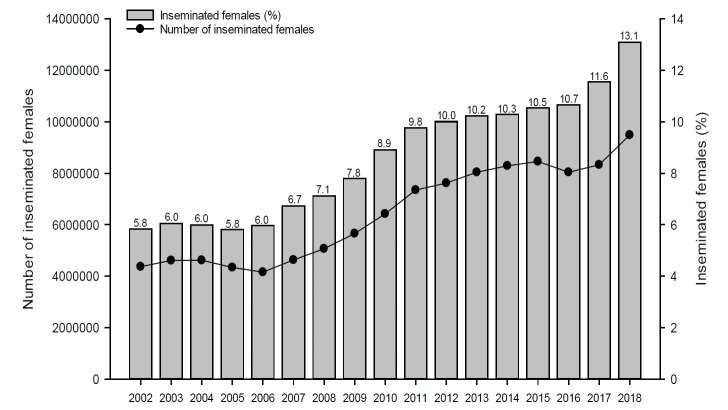
Evolution of the number and percentage of females inseminated in Brazil based on the number of beef and dairy breeding cows and heifers (ANUALPEC, 2018) and the number of marketed doses of semen (ASBIA, 2019). An average of 1.6 inseminations per breeding female was considered.

Based on the number of breeding cows and heifers in Brazil (ANUALPEC, 2018) and the number of commercialized doses of semen (ASBIA, 2019), it was possible to estimate the evolution of the AI market in Brazil from 2002 to 2018 (Fig. 2). In 2002, which coincides with the beginning of data collection (presented in Baruselli *et al*., 2012), only 5.8% of the bovine females of the Brazilian herd were inseminated, taking into account the use of 1.6 doses of semen per breeding female. However, in 2018 this figure reached 13.1% of the total number of females in reproduction of the Brazilian herds (Fig. 2), demonstrating a significant advance in the use of this technology. This increase was mainly due to the use of timed protocols, which are highly reproductive efficient and facilitate the dissemination of artificial insemination. In 2018, it is estimated that approximately 9.5 million females were artificially inseminated in Brazil, contributing to increase the genetic, productive and economic gains of livestock.

Furthermore, statistical data collected in Brazil are similar to those of neighboring countries, according to the information reported by Argentina and Uruguay (2016/2017 breeding season). Uruguay has approximately 3 million breeding females and 300,000 TAI, demonstrating that approximately 10% of breeding females in reproduction are inseminated. In total, more than 15 million breeding females were inseminated using TAI in Brazil, Argentina and Uruguay in the year 2017 (Mapletoft *et al*., 2018). This information indicates the consolidation of the technology in the market, resulting in economic gains and positive perspectives for livestock.

**Figure 3:**
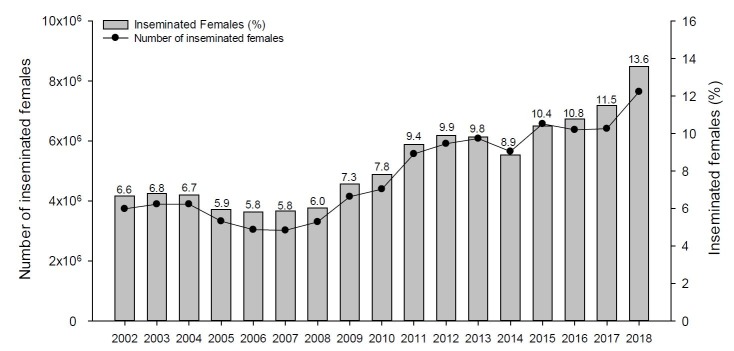
Evolution of the number and percentage of beef females inseminated in Brazil based on the number of beef breeding cows and heifers (ANUALPEC, 2018) and the number of commercialized semen doses (ASBIA, 2019). An average of 1.4 inseminations were considered per breeding female.

**Figure 4:**
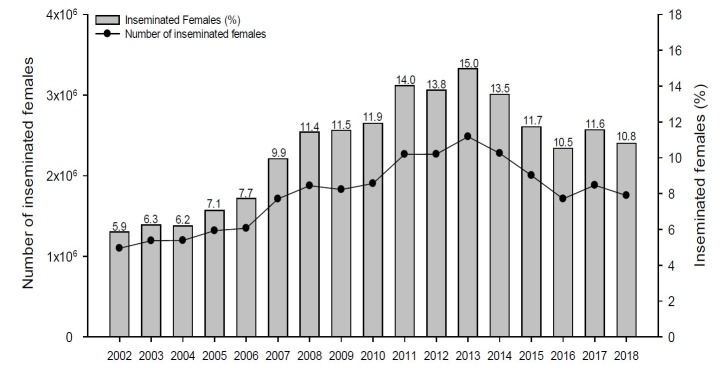
Evolution of the number and percentage of dairy females inseminated in Brazil, based on the number of dairy breeding females (ANUALPEC, 2018) and the number of commercialized doses (ASBIA, 2019). An average of 2.4 inseminations were considered per breeding female.

According to the number of bovine females in reproduction (cows and heifers) in Brazil (ANUALPEC, 2012 and 2018), the percentage of beef (Fig. 3) and dairy (Fig. 4) artificially inseminated females from 2002 to 2018 were calculated. These data were confronted with the number of semen doses commercialized for beef and for dairy herds disclosed by the Brazilian Association of Artificial Insemination (ASBIA, 2019). For the analysis, 1.4 doses of semen per artificially inseminated beef female and 2.4 doses of semen per artificially inseminated dairy females were considered. The data show that 13.6% of the beef females and 10.8% of the dairy females are artificially inseminated in Brazil. Unexpectedly, there was a drop in the percentage of inseminated dairy cows in the last 5 years. On the other hand, the percentage of artificially inseminated beef females has increased almost two times in the last 10 years.

According to preliminary data from 2017 (IBGE), 76.2% of livestock establishments in Brazil had less than 50 head. Since there are many small producers, the low incidence of technology in small farms is proportionately higher, creating a budget constraint for the producer to invest in technology. For dairy cattle, 85.9% of all properties have less than 50 head (IBGE, 2017), which can explain the reduction on the use of AI in the last 5 years (Fig 4). The number of establishments with 50 head or less is also high for beef farms in Brazil (68.1%), however, the number of large properties (441,133 farms, IBGE 2017) is almost 3-fold the number of large dairy properties (164,568), which is indicative of a higher incidence of technology for beef farms. Furthermore, according to the profile of lactating cows in Brazil, 54% of the total is composed of genetics without milk production specialization (Nogueira, 2019). Additionally, in the last 4-years, an increase in the cost of the food for dairy cows was observed and therefore, there was a reduction on the economic gain of the dairy farms (CNA, 2019), discouraging the use of technology in the milk production sector. These facts corroborate with the evolution of the AI market, where it is possible to observe that beef cattle have a growing number of inseminated animals, and dairy cattle, in the last four years (2014 to 2018), have had a decrease on the number of inseminated females.

## The economic impact of TAI in Brazil

In the year of 2018, it is estimated that TAI generated approximately R$ 3.5 billion (approximately 1 billion dollars) for the Brazilian beef and dairy production chain. It is calculated that this activity counts with 3,788 veterinarians specialized in animal reproduction acting on the farms (considering 3,500 TAI per professional, Baruselli, 2019b). Based on these data, it is estimated that TAI moved R$ 796 million for its execution in Brazil. The veterinary service corresponds to 33% of the value for execution of TAI (R$ 265.2 million), considering the cost of R$ 20 per synchronized animal. The companies selling semen and pharmaceuticals represent 66% (R$ 796 million) of the total value, considering 13.3 million TAI performed in the year at an average price of R$ 20 for synchronization drugs and R$ 20 for semen dose (Baruselli, 2019b).

In addition to these direct economic impacts, there are benefits in increasing productivity that must be taken into consideration. In beef herds, there is an increase in the quantity and quality of calves produced with the introduction of TAI technology. Considering that TAI is used in 10.2 million beef females, an increase of 8% is estimated in calf production when compared to natural service (Sá Filho *et al*., 2013, Baruselli *et al*., 2018a), with additional production of 816 thousand calves per year, adding R$ 979 million in the meat production chain (TAI calf price = R$ 1,200.00). Also, because of the high genetic merit and anticipation of the calving provided by the use of TAI comparing to natural service, these studies showed an additional gain of 20 kg in the weaning weight, which represents an extra gain of R$ 490 million (Kg of the calf = R$ 6.00). Also, from weaning to slaughter, calves from TAI show an additional gain of 15 kg of carcass, totaling R$ 600 million (approximately 4 million animals slaughtered with R$ 150.00 per 15 kg of carcass). Thus, TAI generates an impact of additional R$ 2.1 billion per year on the beef production chain when compared to natural service (Baruselli, 2019b).

In dairy herds, TAI also has great economic impact. Studies have shown that there is a reduction of approximately 1 month in the inter calving interval (ICI) of animals receiving TAI when compared to animals submitted to traditional systems of estrous detection or natural mating (Nebel, 2003; Caraviello *et al*., 2006; Teixeira, 2010; Baruselli *et al.*, 2017a). With this reduction of the ICI, there is a 10% increase in the annual milk yield of the farm. Considering that 3.1 million dairy cows are submitted to TAI in Brazil, with an estimated production of 3,000 liters per lactation (estimative for farms that use AI), an increase of 917 million liters of milk per year is estimated, with additional revenues of 1.3 billion additional income per year. In addition, the use of genetically superior bulls through AI adds 350 liters of milk per lactation, corresponding to 131 million liters per year (R$ 1.39 per liter, average of 2018 CEPEA/USP, published in 2019) and a turnover of R$ 166 million per year. Thus, it was estimated that the impact of the TAI on dairy farming generates an additional R$ 1.5 billion per year when compared to traditional breeding systems with estrous detection or natural mating.

From these data it is possible to calculate the return on investment of this biotechnology. Each R$ 1.00 invested on the TAI technology, there is a return of R$ 4.50 for the beef and dairy production chain in Brazil. (Baruselli, 2019b). These figures clearly demonstrate that the investment in TAI technology generates significant gains for Brazilian livestock.

## Evolution of synchronization and resynchronization protocols for TAI

The first TAI protocols for cattle appeared in the mid - 1990s with the development of the Ovsynch protocol (GnRH - 7 days/PGF - 48 hours/GnRH - 16 hours before AI; Pursley *et al*., 1995). Currently, TAI programs underwent various modifications to facilitate management and improve pregnancy rates. In Brazil, the protocol based on estradiol (E2) and progesterone (P4) is the most used for TAI (Baruselli *et al*., 2002; Sá Filho *et al*., 2009; Baruselli *et al*., 2012). Numerous protocols have been developed for different breeds, animal categories and for the producer to adapt the best reproductive program to the farm production system.

Regarding the available TAI programs, many different protocols can be used with similar efficiency. The time of permanence of the P4 device may vary from 5 to 9 days (Bó *et al*., 2007; Baruselli *et al*., 2012, Baruselli *et al*., 2017a; Bo *et al*., 2018). Regarding the number of managements required to perform the synchronization for TAI, there are studies that have developed systems with 3 or 4 animal handlings. In general, the goal of the additional management is to administer prostaglandin (PGF), anticipating luteolysis, and reducing serum P4 concentrations at the end of the protocol in cycling animals. The additional treatment with PGF in cyclic heifers and cows 2 or 3 days before the device removal (four animal handlings) increases the dominant follicle growth and the ovulation and pregnancy rates (Mantovani *et al*., 2005, 2010; Sá Filho *et al*., 2009; Meneghetti *et al*., 2009). However, some studies have demonstrated that it is possible to perform PGF treatment on day zero (D0) of the protocol (3 animal handlings), causing luteolysis in animals with presence of a responsive corpus luteum (CL) at the beginning of synchronization, reducing blood P4 during the protocol, increasing the follicular growth, ovulation and conception and facilitating the management of TAI (Carvalho *et al*., 2008). However, recent studies have shown association between decrease in circulating progesterone concentration during the synchronization protocol and increase of dry matter intake (Batista *et al*., 2015). Therefore, the positive effect of the PGF treatment to induce early luteolysis during the TAI protocol could be reduced in animals submitted to high dry matter intake.

The EB has been successfully used for inducing ovulations at the end of the synchronization protocol (Hanlon *et al*., 1997; Cavalieri *et al*., 2002). Estradiol cypionate (EC) is another ester of E2 with low water solubility. Despite differences in pharmacodynamics, both esters of estradiol (EB and EC) administered either at P4 device removal (EC) or 24 h later (EB) were effective in inducing synchronized LH surge and ovulations and similar P/AI in suckled *Bos indicus* beef cows submitted to TAI (EB = 57.5%; 277/482 vs. EC = 61.8%; 291/471; Sales *et al*., 2012). Furthermore, in lactating Holstein cows the EC administered at P4 device removal (3 animal handlings) or GnRH 48 h later (4 animal handlings) presented similar LH surge (time of the LH peak averaged 43.6 h after P4 device removal) and P/AI (TAI performed 58h after P4 device removal for both treatments, EC = 30.0%; 117/390 vs. GnRH = 31.4%; 123/392; Souza *et al*., 2009). The use of EC as the ovulatory stimulus given at the time of P4 device removal in the TAI protocol reduces the handling, without reducing fertility.

Furthermore, to achieve better genetic and production gains, reproductive strategies should focus on improving service rates and reducing the interval between inseminations, without compromising the viability of the previously established gestation (Sá Filho *et al*., 2014). Based on this concept, ovulation resynchronization protocols were developed for females that did not become pregnant. In these reproductive programs, the non-pregnant females from the previous TAI were identified as soon as possible and inseminated again, therefore increasing the proportion of pregnant cows per AI (Baruselli *et al*., 2017b). This procedure promotes the anticipation of conception in the breeding season; concentrating parturitions at the beginning of the calving season and increasing reproductive efficiency at the subsequent breeding season (Sá Filho *et al*., 2013; Bo and Baruselli, 2014; Bo *et al*., 2016).

Conventional resynchronization is initiated at the time of pregnancy diagnosis (28 to 32 days after TAI; Marques *et al*., 2012; Stevenson *et al*., 2003). With this method, it is possible to perform three inseminations with an 80-days interval. Subsequently, early resynchronization was developed, which is initiated in all females (independently of the pregnancy diagnosis) 22 days after the TAI. On day 30 all females are submitted to pregnancy diagnosis and only the non-pregnant females carry on the TAI protocol, receiving the next artificial insemination on day 32 (Sá Filho *et al*., 2014). With this method, it is possible to perform three inseminations with a 64-days interval. Recently the super-early resynchronization protocol was developed, in which it is possible to perform three inseminations in 48 days. This resynchronization starts in all females 14 days after TAI. On the 22nd, all the non-pregnant females were diagnosed using Doppler ultrasonography (Vieira *et al*., 2014), analyzing the presence and vascular flow of the CL (Pugliesi *et al*., 2017; Siqueira *et al*., 2013).

According to Vieira *et al*. (2014), the application of 1.5 mg of estradiol benzoate (EB) on days 13 to 14 after previous TAI induced luteolysis and reduced conception rate of the first insemination differently from resynchronized females at 22 days post-TAI, which do not present luteolysis after the treatment with estradiol benzoate at the beginning of the resynchronization protocol (Sá Filho *et al*., 2014). Thus, super-early resynchronization is based on the use of injectable P4 at the time of insertion of the P4 device (14 after TAI) to induce the emergence of a new wave of follicular growth (Cavalieri, 2018) without impairing the gestation established in the previous AI (Rezende *et al*., 2016; Guerreiro *et al*., 2018; Gonçales-Junior *et al*., 2018). However, recently Motta *et al*. (2018) reported that 16- to 18-month-old Nellore heifers submitted to super-early resynchronization with 1 mg of EB at day 14 showed early luteolysis, but there was no reduction in the conception rate of the first TAI. This information is indicative of conflicting scientific findings and justify further studies to evaluate the effect of treatment with estradiol at the onset of the super-early resynchronization protocol (Day 14).

## Embryo Transfer Market

The *in vivo* embryo production (*in vivo* derived – IVD) in Brazil had its onset in 1977 (Rubin, 2005), reaching a production of 24,085 embryos in 1997 (IETS, 1998). In the next 7 years, the IVD embryo production increased more 4 fold, with a production of 102,100 embryos in 2004 (IETS 2005). As of this moment, the *in vitro* production (IVP) of embryos, which was implemented in Brazil in 1993 (Rubin, 2005), becomes noticeable, approaching the IVD embryo production (IVD: 102,100 vs. IVP: 80,833). In 2004, 30% of transferred embryos in the world were derived from the IVP of embryos (IVD: 789,000 vs. IVP: 239,813).

In 2007, 46,694 IVD embryos and around 200,000 IVP embryos were transferred in Brazil, clearly showing the exponential growth of the *in vitro* embryo production market (IETS, 2008). At that time, over 90% of these embryos (both IVD and IVP) were produced in beef herds. In 2008, Brazil accounted for 86% of the world production of IVP embryos (IETS, 2009). These data demonstrate that Brazil was a world leader in the use of this technology.

While the IVD embryo production maintained constancy between 2007 (46,694) and 2013 (50,455), the *in vitro* embryo production continued enhancing, reaching its peak production in 2013 with 366,517 IVP embryos (IETS, 2014).

In addition, according to recent data published by the IETS in 2018 (Viana, 2018), there has been an increase of 48.9% in the number of IVP embryos in the world in comparison to the previous year. On the other hand, the number of IVD embryos production had a reduction of 21.7%. Nowadays, the number of IVD embryos produced in the world (495,054) is two times lower than IVP embryos (992,289; Viana, 2018). This increase on the IVP embryos in 2017 occurred mainly in North America (82.7%) and in Europe (164.7%). For the first time since 1999, the number of IVP embryos in North America overcame the number of IVP embryos in South America.

In South America, Brazil and Argentina stand out as the largest bovine herds and largest embryos producers (Viana *et al*, 2018). In 2017, Brazil wasresponsible for the production of 345,528 IVP embryos, which represents 76.2% of all IVP embryos in South America.

In 2017 in Brazil, the IVP embryos was higher for dairy (180.475 embryos) than for beef (165,053 embryos) herds, and in Argentina (2^nd^ in the ranking for IVP embryos in South America), the number of IVP embryos for beef herds was higher than for dairy herds (31,034 vs. 1,710).

In South America the IVD embryo production is of 49,230 embryos, with an average of 5.5 embryos per flushing. Brazil is responsible for approximately 60% of the IVD embryos produced in South America. The second in the ranking is Argentina, comprising 36% of this production. In Brazil, there is a noticeable difference in the proportion of IVD embryos in dairy and beef herds in the last 3 years (Tab. 1). In beef herds, the IVD embryos represents nowadays only 24% of the Brazilian production, the remaining (76%) belongs to the dairy herds. In Argentina, this proportion is reversed, where the beef herds comprise 92% of the IVD embryos market.

**Table 1:** Proportion of IVD and IVP embryos for beef and dairy herds in Brazil from 2015 to 2017.

	*In vivo*			*In vitro*		
	Beef	Dairy	Total embryos	Beef	Dairy	Total embryos
2015	73%	27%	22,355	43%	57%	353,539
2016	48%	52%	31,683	46%	54%	346,817
2017	24%	76%	29,533	48%	52%	345,528

## Evolution of the synchronization and resynchronization protocols for TET

The technique of synchronization of ovulation for timed embryo transfer (TET) significantly increases the amount of recipients suitable for receiving an embryo, making it feasible and easy to use this technology for beef and dairy herds (Baruselli *et al*., 2000; Bo *et al*., 2002; Nasser *et al*., 2004). After synchronization, 75 to 85% of the recipients present a CL that is suitable for ET without the need for estrous detection (Baruselli *et al*., 2010). TET can be used in cyclic or anestrous recipients, optimizing the reproductive efficiency in beef and dairy herds (Rodrigues *et al*., 2010).

In addition to optimizing the dissemination of high value genetic material, the ET improves the reproductive efficiency of repeat-breeder cows and reduces fertility impact caused by heat stress (Baruselli *et al*., 2010). The TET can be used in repeat-breeder cows to increase conception rates mainly during warm periods of the year (Freitas *et al*., 2010, Rodrigues *et al*., 2010; Ferreira *et al*., 2011). In studies conducted in Brazilian tropical conditions, it was observed that the oocyte quality is affected by heat stress during the summer (Torres-Junior *et al*., 2008), which interferes with initial embryo development, especially in repeat-breeder cows (Ferreira *et al*., 2011; Ferreira *et al*., 2016). Studies have indicated that it is possible to obtain a larger numbers of pregnant females in dairy herds with the use of ET during the summer and in repeat-breeder cows.

**Figure 5:**
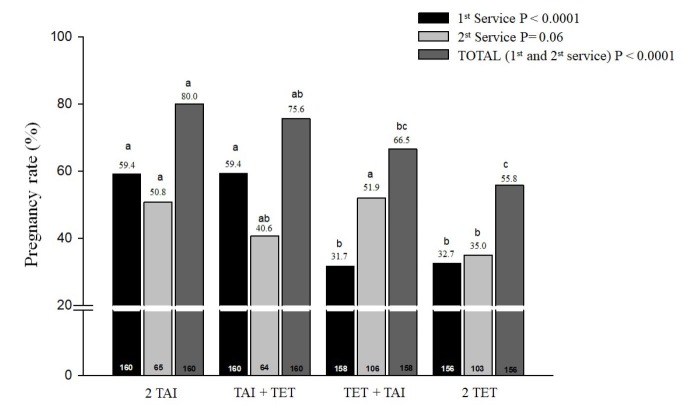
Figure 5. Pregnancy rate of the first and second services and cumulative pregnancy rate (first + second service) in Nelore (*Bos indicus*) lactating cows submited to four reproductive managements: 2TAI (n = 160), TAI followed by TET (TAI/TET; n = 160), TET followed by TAI (TET/TAI; n = 158) and two TET (n = 156). Adapted from Martins *et al*., (2014).

In beef herds, studies evaluated the reproductive efficiency of synchronization and resynchronization techniques used simultaneously for TAI and TET. In one study (Martins et al., 2014), 634 lactating Nellore cows were submitted to four treatments: two consecutive TAI (2TAI; n = 160), TAI followed by TET (TAI/TET; n = 160); TET followed by TAI (TET/TAI; n = 158) and two consecutive TET (2TET; n = 156). The embryos were produced *in vitro* and transferred fresh. The pregnancy rate after the first service was higher for managements with 2TAI (59.4%, 95/160) and TAI + TET (59.4%, 95/160) compared to TET + TAI (31.7%, 50/158) and 2TET (32.7%, 51/156, Figure 5). Furthermore, the accumulated pregnancy rate (1st + 2nd service) was lower for the animals that received 2 TET (P < 0.0001; Fig. 5) when compared to animals that received 2 TAI and TAI+TET. However, no differences were observed between 2 TAI and TAI+TET reproductive programs. These data are indicative of high reproductive efficiency for programs associating TET with TAI.

In order to reduce the negative effects of pregnancy rate using TET, another study was carried out with two resynchronizations (early resynchronization protocol starting 22 days after AI and with diagnosis of gestation at day 30) in Nellore lactating cows (n = 360; unpublished data). The animals received a TET, followed by 2 TAI in a 64-day breeding season. The pregnancy rate for TET was 44.2%, followed by 55.7% for the first TAI (second service) and 47.1% for the second TAI (third service), comprising 86.9% pregnancy rate at the end of 64 days breeding season. These data are indicative that it is possible to use TET on the first service, followed by resynchronizations with TAI, with satisfactory results in the herds breeding season.

Aiming to improve the efficiency of reproductive programs, research groups have studied the use of Doppler ultrasonography (to evaluate the vascularization of the CL) for TET. Several studies have observed the effect of the CL blood flow on embryo recipients pregnancy (Pinaffi, 2015; Pugliesi *et al*., 2016), which allows the use of Doppler ultrasonography to rule out recipients presenting CL without vascularization, directing desirable embryos to more fertile cows (Pugliesi *et al*., 2017).

The possibility of initiating resynchronization after the early pregnancy diagnosis using Doppler was also evaluated. A total of 165 embryo recipients were diagnosed at 21 days, aiming to improve reproductive management of TET programs (Guimarães *et al*., 2015). The accuracy and sensitivity of the early diagnosis at 21 days was of 88.3% and 100%, respectively. This early diagnosis with Doppler enabled 80% of the non-pregnant recipients to be diagnosed at 21 days of gestation and thus resynchronized early for a new TET program.

## Challenges to increase the use of reproductive biotechnologies in Brazil

The markets for AI and ET in Brazil have grown considerably in the last 20 years. The technological development of the reproductive processes has reached high efficiency with favorable cost-benefit when applied in the farms for meat and milk production. However, the challenge to keep the market growing in the next decade could depend on some factors, such as:

- **Extension services for producers:** transfer the scientific and technical knowledge related to increased farm productivity and economic benefits of the application of reproductive biotechnologies on farms, providing a clear message to the users (many farmers do not know the advantages of using reproductive biotechnologies involved with breeding programs).
- **Extension training for specialist:** the educational programs for training new specialists to apply assisted reproductive technologies on farms to support the continuous growth of these technologies (to reach the world average of inseminated cattle in Brazil, 4000 veterinarians working on the farms are necessary).
- **Technological development:** continue to develop more efficient and cost-effective products and practical protocols to apply on the farm to enhance productivity and profitability. Conduct more research to increase the efficiency of AI and ET programs taking into account the interaction between nutrition and reproduction, genetics and reproduction, health and reproduction and environment and reproduction.
- **Increase the integration:** increase the cooperation between universities, research institutes, veterinarians, industries for cattle AI and ET, allied animal production industries, farm associations and producers with the intent of more effectively transferring the use of reproductive technologies to the field.
- **Market demand:** in the world there is a strong demand for production of animal protein with higher efficiency and environmental and economic sustainability. The use of reproductive biotechnologies increases productivity per unit of land and significantly contributes to improve the efficiency of livestock. Therefore with the intensification of the use of reproductive biotechnologies it is possible to enhance production with reduced environmental impact. This scenario contributes to change the production system and for farmers to seek new alternatives, in which reproductive biotechnologies are included.

## Conclusions

The use of reproductive biotechnologies (AI and ET) to multiply superior individuals results in significant genetic advancement of the herds and increased productivity. Despite all the scientific and technological advances that have occurred in recent years, these biotechnologies are still used in small extent in rural properties. Only 13% of Brazilian cows and heifers are artificially inseminated, compared to 20-22% in the world (Thibier and Wagner, 2002; Vishwanath *et al*., 2003). The Brazilian AI market needs to grow 5% per year in the next 10 years to reach the world average of inseminated bovine females. Although Brazil is the largest producer of embryos in the world, the intensity of use of ET is low (number of embryos transferred/total of the herd), ranking the country in the 11th position in the world (Viana *et al*., 2017). Therefore, the Brazilian livestock needs to intensify the use of reproductive biotechnologies for the genetic and productive improvement of the herd. To change this scenario, efficient reproductive programs that aim to increase the use of artificial insemination and embryo transfer on farms have been developed. These programs are currently established and present a positive economic return (Baruselli *et al*., 2018b), generating higher meat and milk production per area and more value for the beef and dairy production chain.

## References

[B001] (ANUALPEC) (2012). Rebanho bovino brasileiro. Sumário..

[B002] (ANUALPEC) (2018). Rebanho bovino brasileiro. Sumário..

[B003] (ABIEC) (2018). Sumário.

[B004] (ASBIA) (2019). Index ASBIA Mercado.

[B005] Baruselli PS, Marques MO, Madureira EH, Bó GA, Neto C, Pinto W, Grandinetti RR (2000). Superestimulação ovariana de receptoras de embriões bovinos visando o aumento de corpo lúteo, concentração de P4 e taxa de prenhez. Arquivos da Faculdade de Veterinária - UFRGS.

[B006] Baruselli PS, Marques MO, Carvalho NAT, Madureira EH, Campos Filho EP (2002). Efeito de diferentes protocolos de inseminação artificial em tempo fixo na eficiência reprodutiva de vacas de corte lactantes. Rev Bras Reprod Anim.

[B007] Baruselli PS, Reis EL, Marques MO, Nasser LF, Bó GA (2004). The use of hormonal treatments to improve reproductive performance of anestrous beef cattle in tropical climates. Anim Reprod Sci.

[B008] Baruselli PS, Ferreira RM, Sá Filho MF, Nasser LFT, Rodrigues CA, Bó GA. (2010). Bovine embryo transfer recipient synchronization and management in tropical environments. Reprod Fertil Dev.

[B009] Baruselli PS, Sales JNS, Sala RV, Vieira LM, Sá Filho MF (2012). History evolution and perspectives of timed artificial insemination programs in Brazil. Anim Reprod.

[B010] Baruselli PS, Ferreira RM, Colli MHA, Elliff FM, Freitas BG (2017). Timed artificial insemination: current challenges and recent advances in reproductive efficiency in beef and dairy herds in Brazil. Anim Reprod.

[B011] Baruselli PS, Marques MO, Borges A, Penteado L (2017). Impactos econômicos do uso de tecnologia reprodutiva na fazenda.

[B012] Baruselli PS, Ferreira RM, Sá Filho MF, Bó GA. (2018). Review: Using artificial insemination v. natural service in beef herds. Animal.

[B013] Baruselli PS, Souza AH, Sá Filho MF, Marques MO, Sales JNS (2018). Genetic market in cattle (Bull, AI, FTAI, MOET and IVP): financial payback based on reproductive efficiency in beef and dairy herds in Brazil. Anim Reprod.

[B014] Baruselli PS (2019). Avaliação do mercado de IATF no Brasil.

[B015] Baruselli PS (2019). IATF gera ganhos que superam R$ 3,5 bilhões nas cadeias de produção de carne e de leite.

[B016] Batista EOS, Macabelli CH, Chiaratti MR, Yasuoka MM, Sala RV, Ortolan MDDV, Jesus EF, Del Valle TA, Rennó FP, Baruselli PS (2015). Impact of bovine genetic group (Bos indicus vs Bos taurus) and level of dry matter intake (high vs. low) on gene expression of liver enzymes related to progesterone metabolism. Anim Reprod.

[B017] Batista EOS, Guerreiro BM, Freitas BG, Silva JCB, Vieira LM, Ferreira RM, Rezende RG, Basso AC, Lopes RNVR, Rennó FP, Souza AH, Baruselli PS (2016). Plasma anti-Müllerian hormone as a predictive endocrine marker to select Bos taurus (Holstein) and Bos indicus (Nelore) calves for in vitro embryo production. Domestic Animal Endocrinology.

[B018] Bó GA, Baruselli PS, Moreno D, Cutaia L, Caccia M, Tríbulo R, Tríbulo H, Mapletoft RJ (2002). The control of follicular wave development for self-appointed embryo transfer programs in cattle. Theriogenology.

[B019] Bó GA, Cutaia L, Peres LC, Pincinato D, Maraña D, Baruselli PS (2007). Technologies for fixed-time artificial insemination and their influence on reproductive performance of Bos indicus cattle. Soc Reprod Fertil Suppl.

[B020] Bó GA, Baruselli PS (2014). Synchronization of ovulation and fixed-time artificial insemination in beef cattle. Animal.

[B021] Bó GA, de la Mata JJ, Baruselli PS, Menchaca A (2016). Alternative programs for synchronizing and resynchronizing ovulation in beef cattle. Theriogenology.

[B022] Bó GA, Huguenine E, Mata JJ, Núñez-Olivera R, Baruselli PS, Menchaca A (2018). Programs for fixed-time artificial insemination in South American beef cattle. Anim Reprod.

[B023] Caraviello DZ, Weigel KA, Fricke PM, Wiltbank MC, Florent MJ, Cook NB, Nordlund KV, Zwald NR, Rawson CL (2006). Survey of management practices on reproductive performance of dairy cattle on large US commercial farms. J Dairy Sci.

[B024] Carvalho JBP, Carvalho NAT, Reis EL, Nichi M, Souza AH, Baruselli PS (2008). Effect of early luteolysis in progesterone-based timed AI protocols in Bos indicus, Bos indicus x Bos taurus, and Bos taurus heifers. Theriogenology.

[B025] Cavalieri J, Coleman C, Rodrigues H, Macmillan KL, Fitzpatrick LA (2002). The effect of timing of administration of oestradiol benzoate on characteristics of oestrus, timing of ovulation and fertility in Bos indicus heifers synchronised with a progesterone releasing intravaginal insert. Aust Vet J.

[B026] Cavalieri J. (2018). Effect of treatment of Bos indicus heifers with progesterone 0, 3 and 6 days after follicular aspiration on follicular dynamics and the timing of oestrus and ovulation. Anim Reprod Sci.

[B027] (CEPEA) (2019). Boletim do leite.

[B028] (CNA) (2019). (CNA).

[B029] Ferreira RM, Ayres H, Chiaratti MR, Ferraz ML, Araújo AB, Rodrigues CA, Watanabe YF, Vireque AA, Joaquim DC, Smith LC, Meirelles FV, Baruselli PS (2011). The low fertility of repeat-breeder cows during summer heat stress is related to a low oocyte competence to develop into blastocysts. J Dairy Sci.

[B030] Ferreira RM, Chiaratti MR, Macabelli CH, Rodrigues CA, Ferraz ML, Watanabe YF, Smith LC, Meirelles FV, Baruselli PS (2016). The infertility of repeat-breeder cows during summer is associated with decreased mitochondrial DNA and increased expression of mitochondrial and apoptotic genes in oocytes. Biol Reprod.

[B031] (FAO) (2017). Cenário da demanda por alimentos no Brasil.

[B032] Freitas BG, Rodrigues CA, Sales JNS, Teixeira AA, Ferreira RM, Ayres H, Ranieri AL, Baruselli PS (2010). Embryonic loss (between 30 and 60 days) followed to artificial insemination or embryo transfer in high production Friesian dairy cattle. Acta Sci Vet.

[B033] Gonçales-Junior WA, Colli MHA, Catussi BLC, Rezende RG, Andrea DL, Castro MW, Freitas BG, Guerreiro BM, Baruselli PS (2018). Effect of treatment with injectable progesterone at the onset of super precocious TAI ressincronization protocol on TAI pregnancy rates of Nellore heifers. Anim Reprod.

[B034] Guerreiro BM, Freitas BG, Colli MHA, Rezende RG, Penteado L, Gonçales-Junior WA, Baruselli PS (2018). P4 serum concentration at the time of TAI in Nelore females treated with 50 vs. 100 mg of injectable P4 (short or long action) in the super precocious resynchronization protocol. Anim Reprod.

[B035] Guimarães CRB, Oliveira ME, Rossi JR, Fernandes CAC, Viana JHM, Palhao MP (2015). Corpus luteum blood flow evaluation on Day 21 to improve the management of embryo recipient herds. Theriogenology.

[B036] Hanlon DW, Williamson NB, Wichtel JJ, Steffert IJ, Craigie AL, Pfeiffer DU (1997). Ovulatory responses and plasma luteinizing hormone concentrations in dairy heifers after treatment with exogenous progesterone and estradiol benzoate. Theriogenology.

[B037] (IBGE) (2017). Efetivo do rebanho brasileiro.

[B038] (IBGE) (2018). Efetivo do rebanho brasileiro.

[B039] (IETS) (1998). Data Retrieval Annual Report.

[B040] (IETS) (2005). Data Retrieval Annual Report.

[B041] (IETS) (2008). Data Retrieval Annual Report.

[B042] (IETS) (2009). Data Retrieval Annual Report.

[B043] (IETS) (2014). Data Retrieval Annual Report.

[B044] (IETS) (2018). Embryo Technology Newsletter.

[B045] Lamb GC, Mercadante VRG (2016). Synchronization and artificial insemination strategies in beef cattle. Vet Clin North Am Food Anim Pract.

[B046] Lima FS, Vries ADE, Risco CA, Santos JEP, Thatcher WW (2010). Economic comparison of natural service and timed artificial insemination breeding programs in dairy cattle. J Dairy Sci.

[B047] Mantovani AP, Reis EL, Gacek F, Bó GA, Binelli M, Baruselli PS (2005). Prolonged use of a progesterone releasing intravaginal device (CIDR^®^) for induction of persistent follicles in bovine embryo recipients. Anim Reprod.

[B048] Mantovani AP, Nichi M, Sá Filho MF, Ayres H, Vettorato LF, Bó GA. (2010). Follicular growth and plasma progesterone patterns in Bos indicus x Bos taurus heifers submitted to different PGF2α/progesterone-based synchronization protocols. Anim Reprod.

[B049] Mapletoft RJ, Bó GA, Baruselli PS, Menchaca A, Sartori R (2018). Evolution of knowledge on ovarian physiology and its contribution to the widespread application of reproductive biotechnologies in South American cattle. Anim Reprod.

[B050] Marques MO, Ribeiro Júnior M, Silva RCP, Sá Filho MF, Vieira LM, Baruselli PS (2012). Ressincronização em bovinos de corte..

[B051] Martins CM, Reis PO, Vieira JH, Soares JG, Vieira LM, Sá Filho MF, Baruselli PS (2014). Effect of association of FTET and TAI in reproductive programs of Nelore females. Anim Reprod.

[B052] Meneghetti M, Sá Filho OG, Peres RFG, Lamb GC, Vasconcelos JLM (2009). Fixed-time artificial insemination with estradiol and progesterone for Bos indicus cows I: basis for development of protocols. Theriogenology.

[B053] Motta IG, Rocha CC, Bissinoto DZ, Arantes G, Junior A, Melo GD, Lafuente BS, Bastos MR, Kleber ML, Madureira EH, Pugliesi G (2018). A novel and safe strategy for resynchronization using estradiol 14 days after timed-ai in beef heifers. Anim Reprod.

[B054] Nasser LF, Reis EL, Oliveira MA, Bó GA, Baruselli PS (2004). Comparison of four synchronization protocols for fixed-time bovine embryo transfer in Bos indicus x Bos taurus recipients. Theriogenology.

[B055] Nebel RL (2003). The key to a successful reproductive management program. Adv Dairy Technol.

[B056] Nogueira MP (2019). Até quando vai o bom momento da pecuária leiteira?.

[B057] Pinaffi FL, Santos ÉS, Silva MGD, Maturana Filho M, Madureira EH, Silva LA (2015). Follicle and corpus luteum size and vascularity as predictors of fertility at the time of artificial insemination and embryo transfer in beef cattle. Pesq Vet Bras.

[B058] Pugliesi G, Silva JCB, Nishimura T, Miyai D, Silva LA, Binelli M (2016). Use of Color-Doppler ultrasonography to improve selection of higher fertility beef recipiente cows for embryo transfer. Anim Reprod.

[B059] Pugliesi G, Rezende RG, Da Silva JCB, Lopes E, Nishimura TK, Baruselli PS, Madureira EH, Binelli M (2017). Uso da ultrassonografia Doppler em programas de IATF e TETF em bovinos. Rev Bras Reprod Anim.

[B060] Pursley JR, Mee MO, Wiltbank MC (1995). Synchronization of ovulation in dairy cows using PGF2α and GnRH. Theriogenology.

[B061] Rezende RG, Freitas BG, Mingoti RD, Colli MHA, Carvalho JBP, Sá Filho MF, Motta JCL, Macedo GG, Baruselli PS (2016). Follicular dynamics of nelore cows submitted to resynchronization 14 days after tai using injectable p4 for synchronization of follicular wave. Anim Reprod.

[B062] Rhodes FM, McDougall S, Burke CR, Verkerk GA, Macmillan KL (2003). Treatment of cows with an extended postpartum anestrous interval. J Dairy Sci.

[B063] Rodgers JC, Bird SL, Larson JE, DiLorenzo N, Dahlen CR, DiCostanzo A, Lam GC (2015). An economic evaluation of estrous synchronization and timed artificial insemination in suckled beef cows. J Anim Sci.

[B064] Rodrigues CA, Teixeira AA, Ferreira RM, Ayres H, Mancilha RF, Souza AH, Baruselli PS (2010). Effect of fixed-time embryo transfer on reproductive efficiency in high-producing repeat-breeder Holstein cows. Anim Reprod Sci.

[B065] Rubin MIB (2005). Histórico dos 20 anos da Sociedade Brasileira de Tecnologia de Embriões (1985-2005). Acta Scientiae Veterinariae.

[B066] Sá Filho MF, Penteado L, Reis EL, Reis TANPS, Galvão KN, Baruselli PS (2013). Timed artificial insemination early in the breeding season improves the reproductive performance of suckled beef cows. Theriogenology.

[B067] Sá Filho MF, Marques MO, Girotto R, Santos FA, Sala RV, Barbuio JP, Baruselli PS (2014). Resynchronization with unknown pregnancy status using progestin-based timed artificial insemination protocol in beef cattle. Theriogenology.

[B068] Sá Filho OG, Meneghetti M, Peres RFG, Lamb GC, Vasconcelos JLM (2009). Fixed-time artificial insemination with estradiol and progesterone for Bos indicus cows II: Strategies and factors affecting fertility. Theriogenology.

[B069] Sales JNS, Carvalho JBP, Crepaldi GA, Cipriano RS, Jacomini JO, Maio JRG, Souza JC, Nogueira GP, Baruselli OS (2012). Effects of two estradiol esters (benzoate and cypionate) on the induction of synchronized ovulations in Bos indicus cows submitted to a timed artificial insemination protocol. Theriogenology.

[B070] Sartori R, Prata AB, Figueiredo ACS, Sanches BV, Pontes GCS, Viana JHM, Pontes JH, Vasconcelos JLM, Pereira MHC, Dode MAN, Monteiro PLJ, Baruselli PS (2016). Update and overview on assisted reproductive technologies (ARTs) in Brazil. Anim Reprod.

[B071] Siqueira LG, Areas VS, Ghetti AM, Fonseca JF, Palhao MP, Fernandes CA, Viana JH (2013). Color Doppler flow imaging for the early detection of nonpregnant cattle at 20 days after timed artificial insemination. J Dairy Sci.

[B072] Souza AH, Viechnieski S, Lima FA, Silva FF, Araujo R, Bo GA, Wiltbank MC, Baruselli PS (2009). Effects of equine chorionic gonadotropin and type of ovulatory stimulus in a timed-AI protocol on reproductive responses in dairy cows. Theriogenology.

[B073] Stevenson JS, Cartmill JA, Hensley BA, El-Zarkouny SZ (2003). Conception rates of dairy cows following early not-pregnant diagnosis by ultrasonography and subsequent treatments with shortened Ovsynch protocol. Theriogenology.

[B074] Teixeira AA (2010). Impacto da Inseminação Artificial em Tempo Fixo na eficiência reprodutiva de vacas de leite de alta produção.

[B075] Thibier M, Wagner HG (2002). World Statistics for artificial insemination in cattle. Livest Prod Sci.

[B076] Torres-Júnior JDS, De FA, Pires M, De Sá WF, Ferreira ADM, Viana JHM, Camargo LSA, Ramos AA, Folhadella IM, Polisseni J, de Freitas C, Clemente CAA, de Sá Filho MF, Paula-Lopes FF, Baruselli PS. (2008). Effect of maternal heat-stress on follicular growth and oocyte competence in Bos indicus cattle. Theriogenology.

[B077] Viana JHM, Figueiredo ACS, Siqueira LGB (2017). Brazilian embryo industry in context: pitfalls, lessons, and expectations for the future. Anim Reprod.

[B078] Viana JHM, Figueiredo ACS, Gonçalves RLR, Siqueira LGB (2018). A historical perspective of embryo-related technologies in South America. Anim Reprod.

[B079] Viana J. (2018). Statistics of embryo production and transfer in domestic farm Animals. A publication of International Embryo Technology Society (IETS). Embryo Technology Newsletter.

[B080] Vieira LM, Sá Filho MF, Pugliesi G, Guerreiro BM, Cristaldo MA, Batista EOS, Freitas BG, Carvalho FJ, Guimaraes LHC, Baruselli PS (2014). Resynchronization in dairy cows 13 days after TAI followed by pregnancy diagnosis based on corpus luteum vascularization by color doppler. Anim Reprod.

[B081] Vishwanath R. (2003). Artificial insemination: the state of the art. Theriogenology.

